# Fuzzy PID control of permanent magnet synchronous motor electric steering engine by improved beetle antennae search algorithm

**DOI:** 10.1038/s41598-024-52600-8

**Published:** 2024-02-05

**Authors:** Bu Zhang, Pingjuan Niu, Xitong Guo, Jiyan He

**Affiliations:** 1 School of Electrical Engineering, Tiangong University, Tianjin, 300387 Tianjin China; 2 School of Electronics and Information Engineering, Tiangong University, Tianjin, 300387 Tianjin China; 3 High Power Semiconductor Lighting Application System Engineering Research Center Ministry of Education, Tiangong University, Tianjin, 300387 Tianjin China; 4 School of Mechanical Engineering, Tiangong University, Tianjin, 300387 Tianjin China; 5 Tianjin Institute of Aerospace Mechanical and Electrical Equipment, Tianjin, 300301 Tianjin China

**Keywords:** Computational science, Computer science, Information technology, Pure mathematics

## Abstract

The accuracy of control in permanent magnet synchronous motor system significantly affects overall mechanical structure safety. To satisfy high-performance control for the position servo of the electric steering engine, this study selects a suitable vector control model for permanent magnet synchronous motor. Additionally, an enhanced beetle antennae search algorithm is designed and employed to optimize the fuzzy proportional–integral–derivative controller. The hybrid fuzzy proportional-integral-derivative controller is then implemented in the control model of the permanent magnet synchronous motor, resulting in the establishment of a novel control model for the electric steering engine driven by the permanent magnet synchronous motor. The test results showed that root-mean-square error of this control model was 0.03 mm and 0.02 mm respectively under the conditions of sinusoidal response, square wave response and step response, which was obviously shorter than all the selected control models. In addition, the standard deviation of the control model designed in this study accounted for less than 4% of root-mean-square error of electric steering engine position under the sinusoidal response condition, so the calculation stability was high. The research results show that the designed control model has a certain reference value for improving servo control performance of permanent magnet synchronous motor.

## Introduction

The electric steering engine control system is a kind of servo system used to control the rudder surface deflection of missiles, flight models and other aircrafts. It mainly includes motor, reducer, controller, etc.^1,2^ with a working principle that the controller receives the external target position signal, calculates the rotating speed and steering direction of the motor through the internal algorithm of the controller, and then outputs the pulse width modulation (PWM) signal to motor. The motor rotates according to duty cycle and frequency of PWM, and transmits rotating force to the motor output shaft through the reducer. The actuator drives the rudder surface to deflect through the rotating motion of the motor. In recent years, some high-end application fields of electric steering engine require electric steering engine to have good dynamic and static response and good anti-interference robustness. The development of electric steering engine has experienced the process from brushless DC motor to brushless DC motor, from analog control to digital control, from traditional reduction gear to planetary gear, harmonic gear and other new reduction gears. At present, research on electric steering engine mainly focuses on reducing volume, improving control accuracy and reliability^3–5^. In terms of control algorithm, proportional-integral-derivative control is the most commonly used method, which makes the system stable and able to respond quickly by adjusting system error. The proportional–integral–derivative control algorithm has advantages of simple structure, easy implementation and strong adaptability, but it also has disadvantages of difficult parameter setting, poor anti-jamming capability and insufficient adaptability to nonlinear system. However, due to the complex structure and high control difficulty of the electric steering engine itself, it is increasingly difficult to meet the requirements for dynamic performance and anti-jamming performance of the electric steering engine in the above fields by using the traditional proportional-integral-derivative controller (PID)^6^. The current mainstream solution is the design and application of nonlinear control algorithms, which is expected to improve dynamic and steady state response of the system. For example, genetic algorithm (GA), sliding mode control (SMC) and fuzzy algorithm (FA), fuzzy PID controller (FPID) is a commonly used solution. However, it is easy to be affected by application environment and the initial parameter stability is poor, so this study attempts to improve the fuzzy PID controller, so as to put forward the intelligent control model for the permanent magnet synchronous motor (PMSM) electric steering engine control system.

The research is divided into four parts. The first part is used to introduce PMSM application status and disadvantages of electric steering engine control system. The second part focuses on the design of the intelligent control model for the control system of the PMSM electric steering engine by beetle antennae search algorithm (BAS) and FPID, which is also the innovation and main contribution of this research. The third step is to use the designed control model to simulate the control of PMSM, and compare experimental results with calculation results of common control models. The fourth part analyzes the simulation results and summarizes the shortcomings of the research.

## Related works

The signal control quality of PMSM is important in safety and service life of overall mechanical structure, so this topic has been studied by a large number of scholars. Cao L et al. proposed an innovative PMSM system speed loop controller—adaptive overtorque sliding mode control method. The main advantage of this method was that there was no need to know the boundary of cumulative disturbance. In this case, severe jitter caused by improper parameter selection can be avoided. Results proved its effectiveness in controlling PMSM system against cumulative interference^7^. He W. et al. systematically studied and compared the PMSM sensorless control system and analyzed the advantages and disadvantages of on-αβ axis reverse method, as well as the impact of on-γδ axis filtering cutoff frequency on estimation error when extracting electromotive force. The final analysis results showed that the position errors were comparable at a certain speed or above^8^. Jiang Q proposed a new PMSM by instruction filtering. This method constructed barrier Liapunov function to ensure that all system states do not violate the constraint boundary, and used instruction filtering technology to solve “complexity explosion” problem in traditional reverse step design. The simulation results showed that PMSM control accuracy of this scheme was higher^9^. Vafaie M H proposed an advanced predictive system including two cascade controllers: the first controller was designed to minimize power loss and the second controller was designed to minimize torque ripple. PMSM motor test results confirmed the effectiveness in reducing power loss and torque ripple^10^. Lalezar et al.^11^ proposed an SMPMSM control method. The experimental test results showed that this method was superior to the traditional predictive current control method and had appropriate dynamic response capacity. Gambi TS presented a high-performance PMSM drive current control technique. This scheme was applied to control loop to realize axes decoupling, counteract interference, and maintain stability under parameter uncertainty. Its development can be directly implemented on microcontrollers and digital signal processors. Results proved its effectiveness and stability^12^.

It can be seen from the previous research that although they have carried out a lot of research on various control tasks in PMSM system, the research on PMSM electric steering engine control is relatively rare, and most of the applied methods are traditional PID control and fuzzy PID controller. This cannot solve the defects of these methods, and these defects will negatively influence control methods performance.

## Design of PMSM electric steering engine control method with improved BAS and fuzzy PID

To improve control accuracy and efficiency of PMSM electric steering engine, this research tries to design an improved BAS algorithm, and uses it to optimize input parameters of fuzzy PID controller, so as to form control model of PMSM electric steering engine. Firstly, selecting the appropriate PMSM mathematical model by actual situation and designing control method based on the classical fuzzy PID are needed.

### PMSM vector control model design based on fuzzy PID control

**Figure 1:**
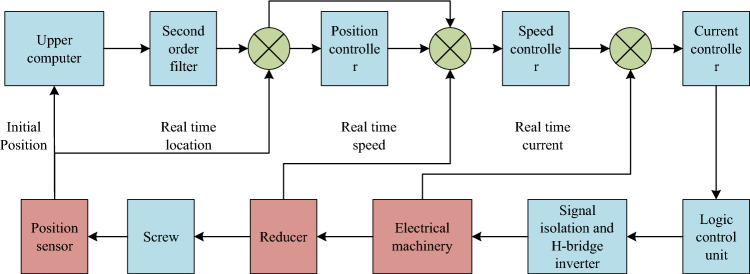
Control block diagram of electric servo system.

In the PMSM system, the electric steering engine controller adjusts PWM signal according to the deviation between actual and target position to make output shaft reach and maintain the target position^13,14^. The electric steering engine control system shall have small size, light weight, fast response, high accuracy and low energy consumption, so as to meet the requirements of actual application. Considering these use requirements, a small electric steering engine control system is designed, and Fig. 1 shows its calculation structure. Upper computer and the position sensor in this control system transmit position information, and click and decelerate to transmit the corresponding real-time current and real-time speed data to the corresponding structure for fusion operation.

PMSM vector control model is a control method based on mathematical model, which can realize accurate control of motor speed, torque and position. There are many factors affecting the true PMSM control process, so the modeling is required to simplify the problem before the control model is applied^15^. The vector control model designed in this study divides the permanent magnet synchronous machine into two parts: stator and rotor. Then, stator and rotor coordinate systems are established respectively. Three-crossing flow is converted into direct flow through coordinate transformation, thus simplifying the control process. The core of vector control model is to regulate the amplitude and phase of stator current through feedback signal, so as to maintain a certain angular relationship with the rotor permanent magnetic field, so as to realize torque control^16,17^. Now, PMSM is selected as the driving part of the electric steering engine to carry out the control task. In the coordinate system $d - q$, the basic PMSM voltage equation is described according to formula (1).

1 $$\left\{ {\begin{array}{*{20}c} {u_{d} = R_{S} i_{d} + P\psi _{d} - \omega \psi _{q} } \\ {u_{q} = R_{S} i_{q} + P\psi _{q} + \omega \psi _{d} } \\ \end{array} } \right.$$

In formula (1), $u_{d}$ and $u_{q}$ are direct and quadrature-axis (DQ) components of stator voltage, $R_{S}$ is stator winding resistance, $P$ is the differential operator, $\omega$ is motor rotor angular frequency, $\psi_{d}$ and $\psi_{q}$ are DQ flux linkages under the rotor coordinate system. Their calculation methods are shown in formula (2).

2 $$\left\{ {\begin{array}{*{20}c} {\psi _{d} = L_{d} i_{d} + \psi _{f} } \\ {\psi _{q} = L_{q} i_{q} } \\ \end{array} } \right.$$

$L_{d}$ and $L_{q}$ are DQ inductances of PMSM; $i_{d}$ and $i_{q}$ are DQ component of stator current; $\psi_{f}$ is the coupling flux link of rotor magnetic steel. PMSM torque equation is shown in formula (3).

3 $$\begin{aligned} T_{e} & = \frac{3}{2}P_{n} \left( {\psi_{d} i_{d} - \psi_{q} i_{q} } \right) \\ & = \frac{3}{2}P_{n} \left[ {\psi_{f} i_{q} + \left( {L_{d} - L_{q} } \right)i_{d} i_{q} } \right] \\ \end{aligned}$$

$P_{n}$ is the PMSM pole pair. DQ inductances can be approximately equal for surface-mounted PMSM. When the direct-axis current is zero in coordinate system, PMSM voltage equation can be expressed using formula (4).

4 $$\left\{ {\begin{array}{*{20}c} {u_{d} = \omega \psi _{q} } \\ {u_{q} = R_{S} i_{q} + P\psi _{q} + \omega \psi _{d} } \\ \end{array} } \right.$$

The electromagnetic torque equation in the model is described according to formula (5).

5 $$T_{e} = \frac{3}{2}P_{n} \psi_{f} i_{q}$$

**Figure 2:**
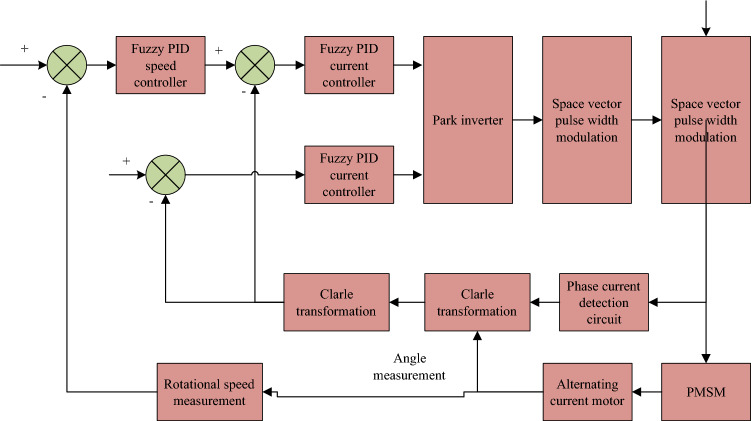
PMSM vector control structure.

At this time, the motor torque is consistent with the quadrature-axis current, which greatly simplifies the control process. Considering the application environment of PMSM system, in this study, the PMSM speed governing system utilizes a dual closed-loop structure, controlling both the rotational speed and current. Fuzzy PID controller is outer loop, while inner loop is current loop controller, which is composed of traditional proportional-integral-derivative (PI) controller. Figure 2 shows the designed PMSM vector control structure.

**Figure 3:**
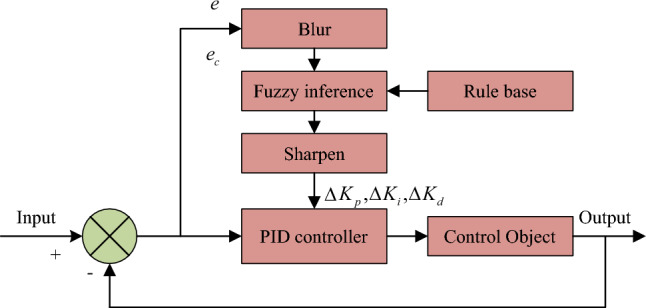
Typical fuzzy PID structure.

Then the appropriate algorithm is selected as the circuit variable control method in Fig. 2. The traditional PID control mode is generally that the current controller and the speed controller adopt PI regulation, which is beneficial to increase the dynamic characteristics. Position controller adopts PID control. Under premise of ensuring dynamic response, the differential control is added to increase dynamic performance. It can adjust control quantity according to the deviation and change rate of the system, so that the system can achieve stable and fast response. However, the core parameters of traditional PID and PI control methods need to be determined manually by the user according to the application environment, resulting in poor control effect of these two methods in some nonlinear time-dependent systems. The fuzzy PID controller is an intelligent control method combining the PID controller and the fuzzy control concept, which can avoid the shortcomings of traditional PID and PI control methods. Figure 3 shows its typical structure.

During the controller operation, it constantly monitors error $e$ and error change rate $e_{c}$, and performs fuzzy processing on them. The controller then deduces according to the fuzzy rules and outputs three parameters $\Delta K_{p}$, $\Delta K_{i}$ and $\Delta K_{d}$, after de-fuzzy, to the PID controller. Then the PID controller parameters $K_{p}$ are adjusted according to formula (6).

6 $$K_{p} = K_{p0} + \Delta K_{p}$$

In formula (6), $K_{p0}$ represents the starting value of $K_{p}$, and $\Delta K_{p}$ respectively represents the change amount of $\Delta K_{p}$. Parameters $K_{i}$ and $K_{d}$ are adjusted in the same way.

### PMSM electric steering engine control model based on improved BAS and fuzzy PID

Although fuzzy PID overcomes the shortcomings of traditional PID, such as insufficient processing capacity for nonlinear and time-dependent system and complex calculation parameter determination process, the selected parameters may not be well adapted to the application environment. Therefore, to further improve control model capability, improved BAS algorithm is designed based on the BAS algorithm with excellent optimization capability, and applied to optimize fuzzy PID calculation parameters in control model. Specifically, PID parameters of the vector controller are adjusted to an appropriate value by using the BAS algorithm, and the PID parameters are corrected in real time by using the fuzzy algorithm. BAS is an intelligent optimization algorithm based on the beetle antennae feeding principle, which can be used to solve function minimization problem. BAS is similar to GA, etc., but the former needs single individual, and the operation amount is much lower than other algorithms. The first step of traditional BAS determines calculation parameters. For the optimization problem of $n$ dimension space, initialization BAS parameters include the number of beetle antennae, left antennae coordinate position $X_{left}$, the right antennae coordinate position $X_{right}$, the center of mass coordinate $X$, the distance between two antennae $D_{0}$, the number of iterations, etc. The second step randomly generates a set of initial input data. As the first generation of beetle antennae, the target function value is calculated corresponding to each beetle antennae, a random vector $Dir = rands(n,1)$ is generated to represent it, and $Dir$ normalization processing is conducted, as shown in formula (7).

7 $$Dir = \frac{{X_{rand} }}{{\left\| {X_{rand} } \right\|}}$$

The third step of the algorithm is to generate a number of new PID parameters as candidate solutions around each beetle antennae according to the step size, and calculate their target function values. Formula (8) is the calculation method.

8 $$X_{left} = X(t) + \frac{{D_{0} Dir}}{2}$$

$X(t)$ represents the center of mass coordinate of $t$ iteration. Formula (9) is calculation method of right margin.

9 $$X_{right} = X(t) - \frac{{D_{0} Dir}}{2}$$

The next step is to select the left and right required values for the fitness function $Fit(X)$ to be optimized. The left required values are shown in formula (10).

10 $$F_{left} = Fit\left( {X_{left} } \right)$$

Formula (11) is calculation method of right margin.

11 $$F_{right} = Fit\left( {X_{right} } \right)$$

Then, the minimum target function value is selected from the candidate solution as the new beetle antennae and replace the original one. If $F_{left} < F_{right}$, then the beetle moves a distance $\Delta x$ in the left direction, which is described in formula (12).

12 $$X(t) = X(t) + \Delta xDir$$

If $F_{left} \ge F_{right}$, then the beetle moves a distance $\Delta x$ in the right direction, which is described in formula (13).

13 $$X(t) = X(t) - \Delta xDir$$

**Figure 4:**
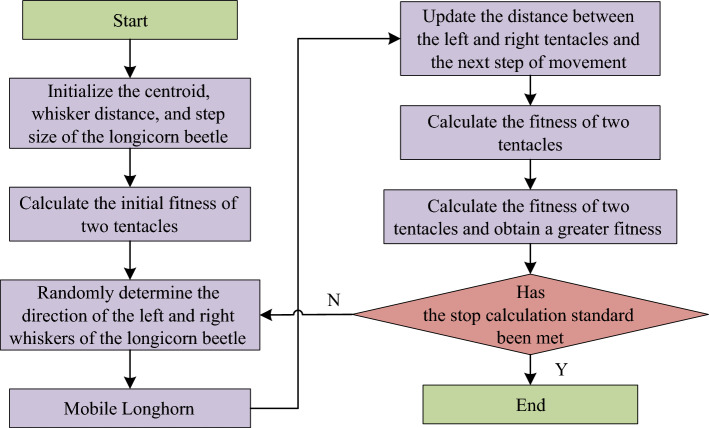
Calculation process of traditional BAS algorithm.

Finally, the above steps are repeated until the preset iteration number is reached or the termination condition is met, the calculation is stopped and the optimal PID parameters are output as the tuning result of the vector controller. Therefore, Fig. 4 shows flow chart of traditional BAS algorithm.

From above, it can be known that the algorithm has the problem of low optimization efficiency, and there is also a contradiction between improving the search accuracy and the search speed. Therefore, the BAS algorithm is now improved. To reduce iterations for BAS algorithm to achieve the required accuracy, the group intelligence idea is introduced. The key to swarm intelligence algorithms is to involve multiple individuals in optimal computing. So, now when the algorithm is initialized, simultaneously define $m$ individual beetle in the $n$ dimension space, and the initial position is randomly distributed. Mass center position of beetle population is represented by a $n$*$m$ matrix, as shown in formula (14).

14 $$X^{k} = \left[ {\begin{array}{*{20}c} {x_{11}^{k} } & {x_{12}^{k} } & \ldots & {x_{1n}^{k} } \\ {x_{21}^{k} } & {x_{22}^{k} } & \ldots & {x_{2n}^{k} } \\ \ldots & \ldots & \ldots & \ldots \\ {x_{m1}^{k} } & {x_{m2}^{k} } & \ldots & {x_{mn}^{k} } \\ \end{array} } \right]$$

In formula (14), $x_{mn}^{k}$ represents the element in the $m$ row and $n$ column after $k$ shifts in the centroid position matrix $X^{k}$. For the conflict between the search accuracy and the search speed in the traditional BAS algorithm, the attenuation coefficient in the original calculation method is cancelled, and the method for calculating the step length and distance in an adaptive manner is designed, so that the step length and the antennae distance in the improved BAS algorithm can be intelligently adjusted according to the corresponding fitness. The adjusted step length $\delta^{k}$ calculation method is shown in formula (15).

15 $$\delta^{k} = \delta_{\max } - \left( {\delta_{\max } - \delta_{\min } } \right)\left( {1 - e^{{ - \beta \left( {\frac{{fit_{k} }}{{fit_{t} }}} \right)}} } \right)$$

In formula (15), $\delta_{\max }$ and $\delta_{\min }$ are the parameters set according to the research problem, $fit_{k}$ is the current fitness value of beetle, $fit_{t}$ represents the target fitness value, and $\beta$ is a constant greater than 10. Formula (16) is the adjusted distance $l^{k}$ between contacts.

16 $$l^{k} = l_{\max } - \left( {l_{\max } - l_{\min } } \right)\left( {1 - e^{{ - \beta \left( {\frac{{fit_{k} }}{{fit_{t} }}} \right)}} } \right)$$

**Figure 5:**
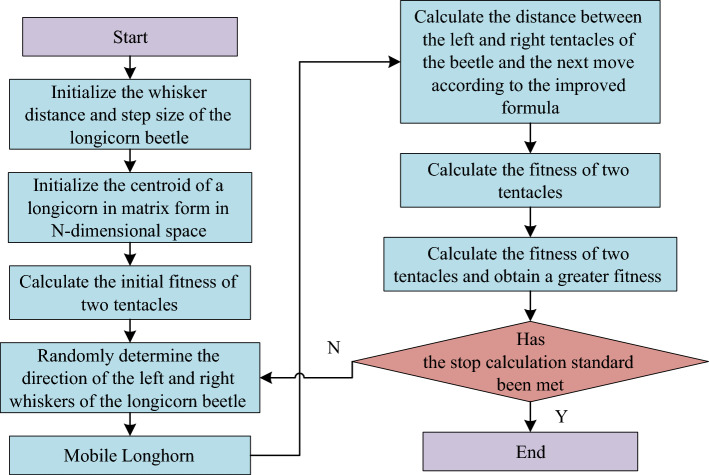
Calculation flow of the improved BAS algorithm.

In formula (16), $l_{\max }$ and $l_{\min }$ are also the parameters set according to the research question. To sum up, Fig. 5 shows calculation flow of improved BAS algorithm.

**Figure 6:**
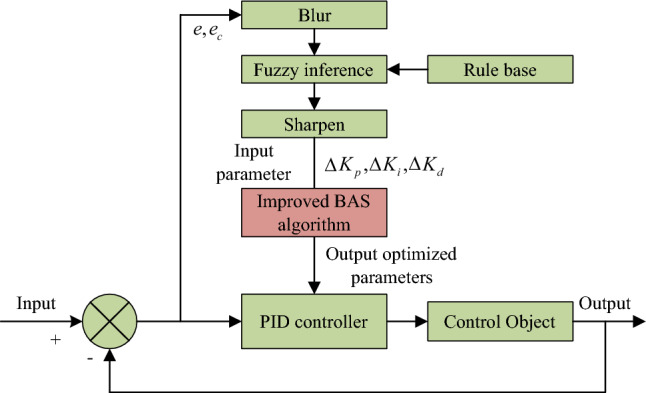
Calculation structure of fuzzy PID controller based on hybrid improved BAS algorithm.

So far, a fuzzy PID controller with hybrid improved BAS algorithm can be obtained. Its calculation structure is shown in Fig. 6. The control effect of fuzzy PID algorithm will be greatly affected by the numerical values of differential, integral and proportional coefficients. Therefore, when the fuzzy PID controller is affected by external interference, the dynamic performance will change significantly. Here, directly input the clear parameters into the improved BAS algorithm, and the parameters of the fuzzy PID can be obtained through the training of the improved BAS algorithm.

Finally, by replacing fuzzy PID module in PMSM vector control model with fuzzy PID controller of hybrid improved BAS algorithm, the PMSM electric steering engine control model required for this study can be obtained. However, to test control model performance, a simulation test experiment shall be designed and conducted.

## Improved PMSM electric steering engine control model application effect test

The reference value of the model cannot be proved only by theoretically designing the PMSM electric steering engine control model of the hybrid improved BAS algorithm and the fuzzy PID controller. Therefore, MATLAB simulation software builds PMSM vector control model, and simulation test is conducted by setting three electrical signals, i.e. step response, sinusoidal response and square wave response. The frequency of sinusoidal response is usually 5 Hz.

### Experiment scheme design

The parameter selection scheme of the control model obtained after multiple debugging is as follows: the maximum iteration number is 120, the initial length of the beetle antennae is 10, the step attenuation factor is 0.95, $\delta_{\max }$ and $\delta_{\min }$ are 0.52 and 0.13 respectively, $l_{\max }$ and $l_{\min }$ are 1.20 and 0.33 respectively. The initial motor rotating speed is 400 r/min, and rotating speed increases to the PMSM level after two seconds of rotation. Table 1 shows the selected PMSM motor parameters. In addition, by comparing control model performance of this design, the other three control models are constructed by selecting fuzzy PID, Fast RCNN algorithm and fuzzy PID algorithm integrating BAS to carry out parallel simulation test. Note that the fusion method of BAS in the fuzzy PID algorithm fused with BAS is the same as the method designed in this study, except that the BAS algorithm in this comparison method is not improved. Mean square error (MSE) and root mean square error (RMSE) control time are selected to evaluate model performance. The parameters of other comparison models are also obtained according to multiple trial runs.

**Table 1:** Core parameters of PMSM.

Number	Parameter name	Unit	Parameter value	Number	Parameter name	Unit	Parameter value
#01	Quadrature axis inductance	mH	0.00570	#05	Permanent magnet flux linkage	Wb	0.1230
#02	Stator resistance	Ω	1.33	#06	Moment of inertia	kg	0.00322
#03	Direct-axis inductance	mH	0.00570	#07	Dc bus voltage	V	308
#04	Power	kW	1.50	#08	Number of poles	/	4

### Experimental results analysis

**Figure 7:**
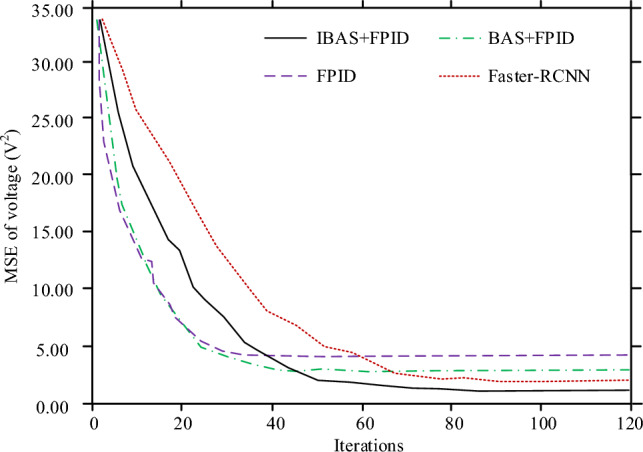
Voltage MSE changes of each control model in the iterative process.

Firstly, the MSE value of each control model is analyzed within one second in the iterative process, as shown in Fig. 7. The horizontal axis and vertical axis are respectively used to describe the iteration times of each control model and the voltage control MSE operating for 1 s in each stage of the iterative process. In Fig. 7, from the perspective of training speed, training speed of control model (IMPROVED BAS + PID, IBAS + PID) designed in this study is between BAS + PID and Fast RCNN model, and it converges when the iteration times reach about 63. From the perspective of training effect, when all control models are trained, the voltage MSE values of IBAS + PID, BAS + PID, FPID and Faster-RCNN models are 1.45V^2^, 3.51V^2^, 4.83V^2^ and 2.14V^2^ respectively.

**Figure 8:**
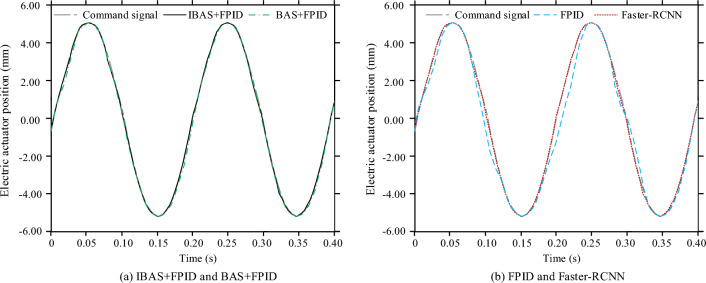
Position changes of electric stepping gear under sinologic response of each control model.

The control effect of the control model completed by each training under three different corresponding conditions of sine, square wave and step is compared as follows. First, analyze the control effect under the sine response of 5 Hz frequency, as shown in Fig. 8. Note that the vertical axis in Fig. 8 is the position of the electric steering engine, and the horizontal axis is the time after the test is started, in s. In Fig. 8, there are different degrees of errors between each control model and the command signal, but the FPID control model has the largest deviation. Taking RMSE as the analysis index, the sinusoidal response electric steering engine positions RMSE of IBAS + PID, BAS + PID, FPID and Faster-RCNN models are 0.03 mm, 0.06 mm, 0.26 mm and 0.05 mm respectively.

**Figure 9:**
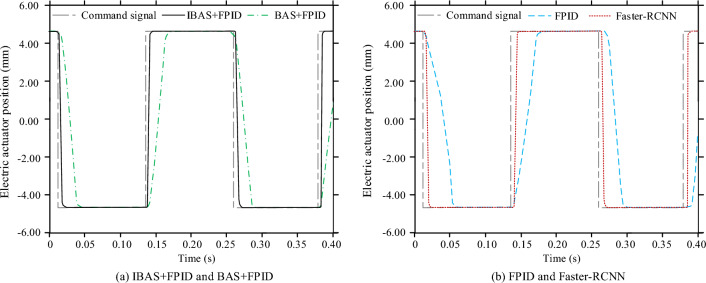
Position changes of electric stepping gear of each control model under square wave response.

The control effects of the control models completed by the training under the square wave response are compared below. The meaning of the sub-map distribution and graphic elements in Fig. 9 is the same as that in Fig. 8. There are still different degrees of errors between each control model and the command signal, but the IBAS + PID control model has the minimum deviation and the fastest response to the position change of the electric steering engine. However, the control accuracy of Fast RCNN model is slightly different from that of IBAS + PID model, but the response speed is slower. Taking RMSE as the analysis index, the square wave response electric steering engine positions of IBAS + PID, BAS + PID, FPID and Faster-RCNN models are 0.02 mm, 0.23 mm, 0.73 mm and 0.03 mm respectively.

**Figure 10:**
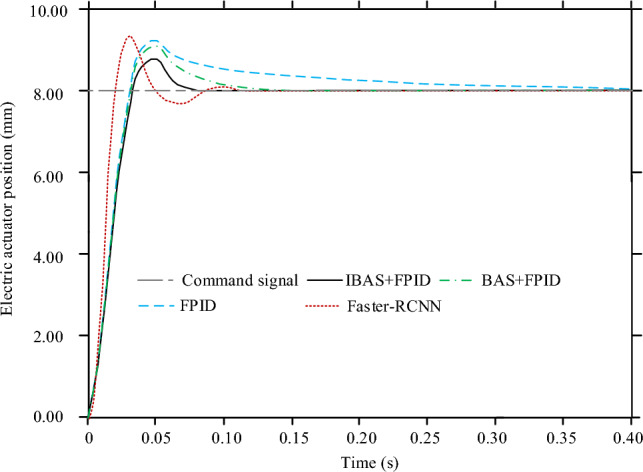
Position changes of electric stepping gear of each control model under step response.

Then compare the control effect of the control model completed by each training under the step response, as shown in Fig. 10. Although each control model is moving towards the command signal, their control trajectory varies considerably. At the beginning of each control model, the feedback electric steering gear position increases rapidly with time, and slows down the growth rate after exceeding the command signal until it changes to the descending electric steering gear position. However, the FPID model's pullback takes the longest time to reach the command signal position amount, and there is no second overshoot. The BAS + PID control model also completes the callback through monotonic decrement. The difference from the FPID control model is that the time required for the callback of the former model is greatly shortened. Faster-RCNN control mode pullback takes less time to reach the command signal for the first time, but multiple pullbacks occur. The whole control process of the IBAS + PID control system is the shortest, and there is no repeated fluctuation and pullback phenomenon. The time consuming for IBAS + PID, BAS + PID, FPID and Fast RCNN control models to complete step response signal control is 0.07 s, 0.13 s, 039 s and 0.11 s respectively.

Finally, the stability of each control model in the control process is analyzed. The stability includes the RMSE stability of the electric steering gear position in the control period of 1 s and the stability of the control time. Each calculation index is shown in the form of mean ± standard deviation. Note that the data for the position of the electric steering engine is calculated under the most representative sine effect conditions. In Table 2, stability of IBAS + PID control model and the Fast-RCNN control model designed in this study is obviously superior to the rest of the models, and the stability difference between the two models is small. To be specific, the ratio of RMS mean value of electric steering engine position and standard deviation of control time of IBAS + PID and Fast-RCNN control models in the four operation times schemes to the corresponding mean value is not more than 4%, while for BAS + PID and FPID control models, the ratio is within the range of 8.0–2.0%

**Table 2:** Comparison of control process stability data of each control model.

Index (index)	Run times	IBAS + PID	BAS + PID	FPID (FPID)	Fast RCNN
RMSE for electric stepping gear position (μ M)	10	32.0 ± 1.1	63.5 ± 5.2	261.5 ± 29.2	51.9 ± 1.7
	100	31.8 ± 1.2	60.8 ± 4.2	235.1 ± 31.6	51.4 ± 1.9
	1000	31.9 ± 1.1	61.4 ± 7.6	274.3 ± 30.8	51.6 ± 2.0
	10,000	32.0 ± 1.1	64.3 ± 8.2	264.9 ± 22.5	51.8 ± 2.0
Control time (ms)	10	71.5 ± 2.4	± 14.6	394.2 ± 58.1	111.6 ± 3.2
	100	71.2 ± 2.6	127.4 ± 18.2	342.6 ± 82.6	113.1 ± 2.8
	1000	71.9 ± 2.8	136.2 ± 14.7	385.4 ± 62.3	114.7 ± 3.0
	10,000	71.6 ± 2.5	129.0 ± 20.4	425.2 ± 94.1	12.5 ± 2.9

## Conclusion

In this study, a new control algorithm for small electric steering engine is designed, which is by the fuzzy PID controller and uses the improved BAS algorithm to optimize the fuzzy PID parameters. To test control model performance, a PMSM simulation control test based on the traditional three-closed loop structure is designed. The test results showed that when all control models were trained, the voltage MSE values of IBAS + PID, BAS + PID, FPID and Faster-RCNN models were 1.45V2, 3.51V2, 4.83V2 and 2.14V2 respectively. When a sinusoidal response was used for the test, there were varying degrees of error between each control model and the command signal, but the FPID control model was the most biased. Taking RMSE as the analysis index, the sinusoidal response electric steering gear positions RMSE of IBAS + PID, BAS + PID, FPID and Faster-RCNN models were 0.03 mm, 0.06 mm, 0.26 mm and 0.05 mm respectively. When testing with square wave response, the control deviation of IBAS + PID control model was the smallest and the response to electric steering gear position change as the fastest. However, the control accuracy of Fast RCNN model was slightly different from that of IBAS + PID model, but the response speed was slower. Taking RMSE as the analysis index, the square wave response electric steering engine positions of IBAS + PID, BAS + PID, FPID and Faster-RCNN models were respectively 0.02 mm, 0.2 mm, 0.73 mm and 0.03 mm. When the test was conducted with step response, the whole control process of the IBAS + PID control system designed in this study had the shortest time consumption and no repeated fluctuation and pullback phenomenon occurred. The time consuming for IBAS + PID, BAS + PID, FPID and Fast RCNN control models to complete step response signal control was 0.07 s, 0.13 s, 039 s and 0.11 s respectively. Standard deviation ratio of mean value of the electric steering engine position RMSE and the control time consumption of the IBAS + PID and Fast RCNN control models in the four operation times schemes to the corresponding mean value was not more than 4%, and the control stability of the two models was relatively better. However, the disadvantage of this study is that, due to the limited research conditions, the designed control method cannot be arranged in the PMSM product system for physical test, which is also the aspect to be improved in the follow-up research.

## Data Availability

All data generated or analysed during this study are included in this published article.
